# Measurement of Environmentally Influenced Variations in Anthocyanin Accumulations in *Brassica rapa* subsp. *Chinensis* (Bok Choy) Using Hyperspectral Imaging

**DOI:** 10.3389/fpls.2021.693854

**Published:** 2021-08-19

**Authors:** Hyo-suk Kim, Ji Hye Yoo, Soo Hyun Park, Jun-Sik Kim, Youngchul Chung, Jae Hun Kim, Hyoung Seok Kim

**Affiliations:** ^1^Sensor System Research Center, Korea Institute of Science and Technology (KIST), Seoul, South Korea; ^2^Department of Electronics and Communications Engineering, Kwangwoon University, Seoul, South Korea; ^3^Smart Farm Convergence Research Center, Korea Institute of Science and Technology (KIST), Gangneung, South Korea; ^4^Center for Intelligent and Interactive Robotics, Korea Institute of Science and Technology (KIST), Seoul, South Korea

**Keywords:** bok choy, health promotion, non-destructive, image analysis, indoor farming

## Abstract

Dietary supplements of anthocyanin-rich vegetables have been known to increase potential health benefits for humans. The optimization of environmental conditions to increase the level of anthocyanin accumulations in vegetables during the cultivation periods is particularly important in terms of the improvement of agricultural values in the indoor farm using artificial light and climate controlling systems. This study reports on the measurement of variations in anthocyanin accumulations in leaf tissues of four different cultivars in *Brassica rapa* var. *chinensis* (bok choy) grown under the different environmental conditions of the indoor farm using hyperspectral imaging. Anthocyanin accumulations estimated by hyperspectral imaging were compared with the measured anthocyanin accumulation obtained by destructive analysis. Between hyperspectral imaging and destructive analysis values, no significant differences in anthocyanin accumulation were observed across four bok choy cultivars grown under the anthocyanin stimulation environmental condition, whereas the estimated anthocyanin accumulations displayed cultivar-dependent significant differences, suggesting that hyperspectral imaging can be employed to measure variations in anthocyanin accumulations of different bok choy cultivars. Increased accumulation of anthocyanin under the stimulation condition for anthocyanin accumulation was observed in “purple magic” and “red stem” by both hyperspectral imaging and destructive analysis. In the different growth stages, no significant differences in anthocyanin accumulation were found in each cultivar by both hyperspectral imaging and destructive analysis. These results suggest that hyperspectral imaging can provide comparable analytic capability with destructive analysis to measure variations in anthocyanin accumulation that occurred under the different light and temperature conditions of the indoor farm. Leaf image analysis measuring the percentage of purple color area in the total leaf area displayed successful classification of anthocyanin accumulation in four bok choy cultivars in comparison to hyperspectral imaging and destructive analysis, but it also showed limitation to reflect the level of color saturation caused by anthocyanin accumulation under different environmental conditions in “red stem,” “white stem,” and “green stem.” Finally, our hyperspectral imaging system was modified to be applied onto the high-throughput plant phenotyping system, and its test to analyze the variation of anthocyanin accumulation in four cultivars showed comparable results with the result of the destructive analysis.

## Introduction

Anthocyanin, a group of water-soluble flavonoid pigments derived from the phenylpropanoid pathway, is responsible for the color of different plant tissues, such as flower, fruit, and leaf ranging from red to violet and blue (Strack and Wray, 1989). Recent evidence suggests that the dietary supplements of anthocyanin-rich vegetables are closely associated with the reduced risk of cardiovascular disease and cancer (Williams et al., 2004; Butelli et al., 2008; de Pascual-Teresa et al., 2010). Because of the eye-catching color and potential human health beneficial effects of anthocyanin, the improvement of anthocyanin accumulation in plants was attempted through the selection of high-anthocyanin germplasm and the optimization of cultivation environments including light and temperature (Bian et al., 2015; Passeri et al., 2016).

Since anthocyanin accumulations varied during the growing periods of the plant depending on the plant genetic background, environmental conditions, and physiological stress (Chalker-Scott, 1999; Sibley et al., 1999), the non-destructive method of anthocyanin content estimation would be extremely valuable and especially useful for the investigations of pigment changes in individual intact leaves over time. As the less expensive approach, color leaf images obtained by the commercial digital camera can also be considered as an alternative parameter for non-destructive anthocyanin estimation (Simko et al., 2016). The hyperspectral imaging technique is an advanced imaging technology that can combine the advantages of spectroscopic and imaging techniques to detect the continuous wavelengths from visible to near-infrared lights selectively. Hyperspectral imaging has been successfully employed in previous studies to measure anthocyanin contents in various vegetables and fruits in a non-destructive manner (Liu et al., 2017; Gabrielli et al., 2021). However, it has been rarely tested to measure differentially expressed anthocyanin in plants grown under different environmental conditions during the growing period of vegetables.

The objective of this study was to determine that hyperspectral imaging and commercially available color imaging techniques could be employed to analyze the variations in anthocyanin accumulation and be differentially expressed in leaf tissues by cultivar-dependent genetic effect, influence of environmental factors, and their genetic × environmental (G × E) associations. The target plant was a bok choy (*Brassica rapa* var. *chinensis*), an important dietary vegetable cultivated and consumed worldwide for its edible leaves, and cultivation experiments were performed in an indoor farm with LED artificial light climate control systems.

## Materials and Methods

### Plant Materials and Experimental Design

The first experiment was conducted to select and calibrate proper anthocyanin reflectance indices, such as anthocyanin absorption index (Merzlyak et al., 2003) and three bands model (Gitelson et al., 2009) in bok choy. Four commercial cultivars that had the variation in anthocyanin accumulations, “green stem,” “white stem,” “red stem,” and “purple magic” (Asia Seed Co., Republic of Korea) were used in this experiment. Seeds of each bok choy cultivar were germinated in small pots filled with a horticultural soil mix (Nongwoo Co., Republic of Korea). Seedlings of 3-week-old were transplanted into 10 cm pots and grown in a growth room at the Korea Institute of Science and Technology at Gangneung under a 28 /20°C and 14/10 h day/night temperature regime and with LED artificial light (110 μmol/m^2^/s). After 6 weeks of growing, the third and fourth leaves from apical meristem in each plant were subjected to hyperspectral imaging and collected for chemical analysis.

The second experiment was conducted to determine whether hyperspectral imaging and commercially available color imaging techniques could be employed to analyze variations in anthocyanin accumulation, differentially expressed in leaf tissues by cultivar-dependent genetic effect, the influence of environmental factors, and their genetic × environmental (G × E) associations. For the second experiment, the same cultivars were subjected to two different environmental conditions (different light intensity, temperature, and cultivation period) after seedling. The environmental condition for relatively lower light intensity and slight temperature differences (NC; non-stimulation condition) was the same as the environmental condition for the first experiment. The environmental condition for relatively higher light and extreme temperature difference (SC; stimulation condition) was at a growth room under a 28/15°C and 14/10 h day/night temperature regime and with LED artificial light (300 μmol/m^2^/s). The leaf samples were subjected after 2 (GS1) and 4 (GS2) weeks of growing for hyperspectral imaging and then collected for color image acquisition and chemical analysis. Leaf color images were taken together with the reference color card (CTrax 24ColorCard-2 × 3, Camera Trax, Las Vegas, NV, United States).

The third experiment was performed to confirm the possibility of hyperspectral imaging to be applied into the “sensor-to-plant”-type high-throughput phenotyping stage (Lee et al., 2018) for the measurement of anthocyanin accumulations in the whole plant level. The same cultivars were prepared under the stimulation condition for anthocyanin accumulation (SC), and 2 week-grown whole plant (GS1) was subjected to hyperspectral imaging.

The experimental design was a randomized complete block with four replicates of five plants.

### Hyperspectral Imaging Data Acquisition

**Figure 1:**
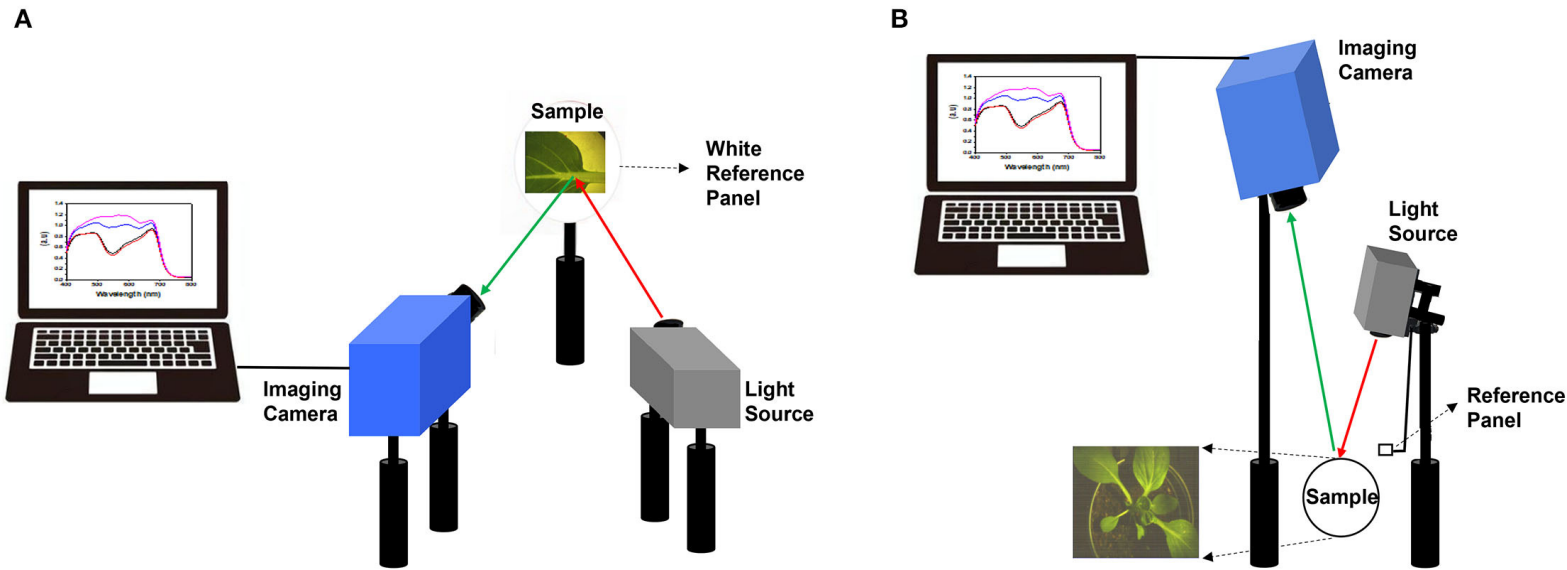
Schematic diagram of hyperspectral imaging employed for target leaf analysis in the first and second experiment **(A)**, and whole plant analysis in the third experiment **(B)**.

Bok choy leaves and whole plants were imaged with SOC710-VP hyperspectral imager (Surface Optics Corp., San Diego, CA, United States). The hyperspectral imager is composed of 520 lines, 696 samples, and 128 bands (the spectral resolution of 4.69 nm). For the first and second experiments, the target leaf was placed at the focal plane of a hyperspectral imaging system, consisting of a hyperspectral camera and halogen lamp in the same growth room. The distance from the target leaf was about 30 cm for both the hyperspectral imaging camera and the light source. The hyperspectral image was acquired by placing a leaf on a white reference panel to distinguish the leaf from the background (Figure 1A).

For the third experiment, whole plant hyperspectral images were acquired as top view with the hyperspectral camera mounted 100 cm above the target plant in the same growth room. Halogen lamp was mounted 50 cm above the target plant to irradiate light, and a separate white reference panel was added to the light source part (Figure 1B).

After hyperspectral image scanning, we calculated transmittance and reflectance spectra of white reference panel and target samples. All hyperspectral imaging experiments were performed under the conventional indoor light environment. In addition, a white panel was adopted to monitor indoor light conditions, including irradiating halogen lamps. To minimize the light-affected environmental condition, we applied the calibration curve obtained from response correction to hyperspectral images so that the obtained spectra are localized only to leaves.

### Chemical Measurement of Anthocyanin Accumulation

Anthocyanin accumulation in leaf tissue was analyzed as described in Gitelson et al.'s study (2001). Freeze-dried tissues of 0.5 g were homogenized in 10 ml of 70% methanol and passed through a paper filter after 5 min of sonication. Distilled water was then added to equal 0.2 of the extract volume, and the diluted filtrate was centrifuged in glass test tubes for 10 min at 3,000 g to separate the water–methanol phases. The absorption spectra of the water–methanol fraction were calculated using a plant reader (Epoch, BioTek Instruments Inc., Winooski, VT, United States). Anthocyanin accumulations were normalized to dry weight (mg/g).

### Selection and Optimization of Reflectance Index Model to Estimate Anthocyanin Accumulation in Bok Choy

Gitelson et al. (2009) established a non-destructive technique to estimate anthocyanin contents in leaves of various tree species using various indices, such as absorbance (abs) value for anthocyanin, anthocyanin reflectance index (ARI), modified anthocyanin reflectance index (mARI), and anthocyanin content index (ACI). Among these indices, mARI is a three-band model based on measurements of tissue reflectance (R) at the specific wavelengths obtained from hyperspectral imaging: relative anthocyanin accumulation [(R759.5–797.02) × (1/R550.14–1/R701.06)] to present the actual and precise wavelengths and anthocyanin values (Merzlyak et al., 2003; Gitelson et al., 2006). We slightly modified the wavelength of this equation to correct the wavelength mismatch and visualize the precise anthocyanin value. The modified equation is [(R759.5–797.02) × (1/R550.14–1/R706.35)]. Using these previously developed anthocyanin estimation indices with our wavelength-corrected mARI, we analyzed the linear relationship between hyperspectral imaging and destructive analysis values to select the optimum index for anthocyanin estimation in bok choy. Total 20 plant samples obtained from the first experiment were subjected to both hyperspectral imaging and destructive analysis. As the result, the highest linear relationship between the destructive and non-destructive (hyperspectral imaging) of anthocyanin content was wavelength-corrected mARI (*R*^2^ = 0.9958), followed by mARI (*R*^2^ = 0.8795), abs value (*R*^2^ = 0.8115), ARI (*R*^2^ = 0.4295), and ACI (*R*^2^ = 0.0224). Therefore, our wavelength-corrected mARI was selected as the anthocyanin reflectance index in bok choy, and anthocyanin accumulation expressed on a leaf area basis (mg/cm^2^) were converted to dry weight (mg/g) using the following formula obtained from the data set of the first experiment: *y* = 1.8123*x* + 0.0962 (*R*^2^ = 0.9958) (Supplementary Figure 1). This anthocyanin measurement method was applied to the hyperspectral imaging-based analysis of anthocyanin variations in the second and third experiments.

### Leaf Image Analysis

All images of four cultivars acquired with the reference color card were corrected and auto-segmented from the background, and their color area was analyzed using the Leaf Analysis tool (NOROO KIBAN Systems Inc., Seongnam, Republic of Korea). We analyzed the area occupied by red and purple colors (RGB ± 20%) in the leaf area as percentages, separately (**Figure 4A**).

### Statistical Analysis

All data were represented as means ± SD of at least four independent experiments. Statistical analysis was carried out using the SAS 9.4 software (SAS Institute Inc., Cary, NC, United States). The ANOVA was the Fisher's Least Significant Difference (LSD) analysis based on the 0.05 probability level.

## Results

### Comparison of Anthocyanin Accumulations Estimated by Hyperspectral Imaging With the Value Measured by Wet Chemical (Destructive) Assay in Different Bok Choy Cultivars

**Figure 2:**
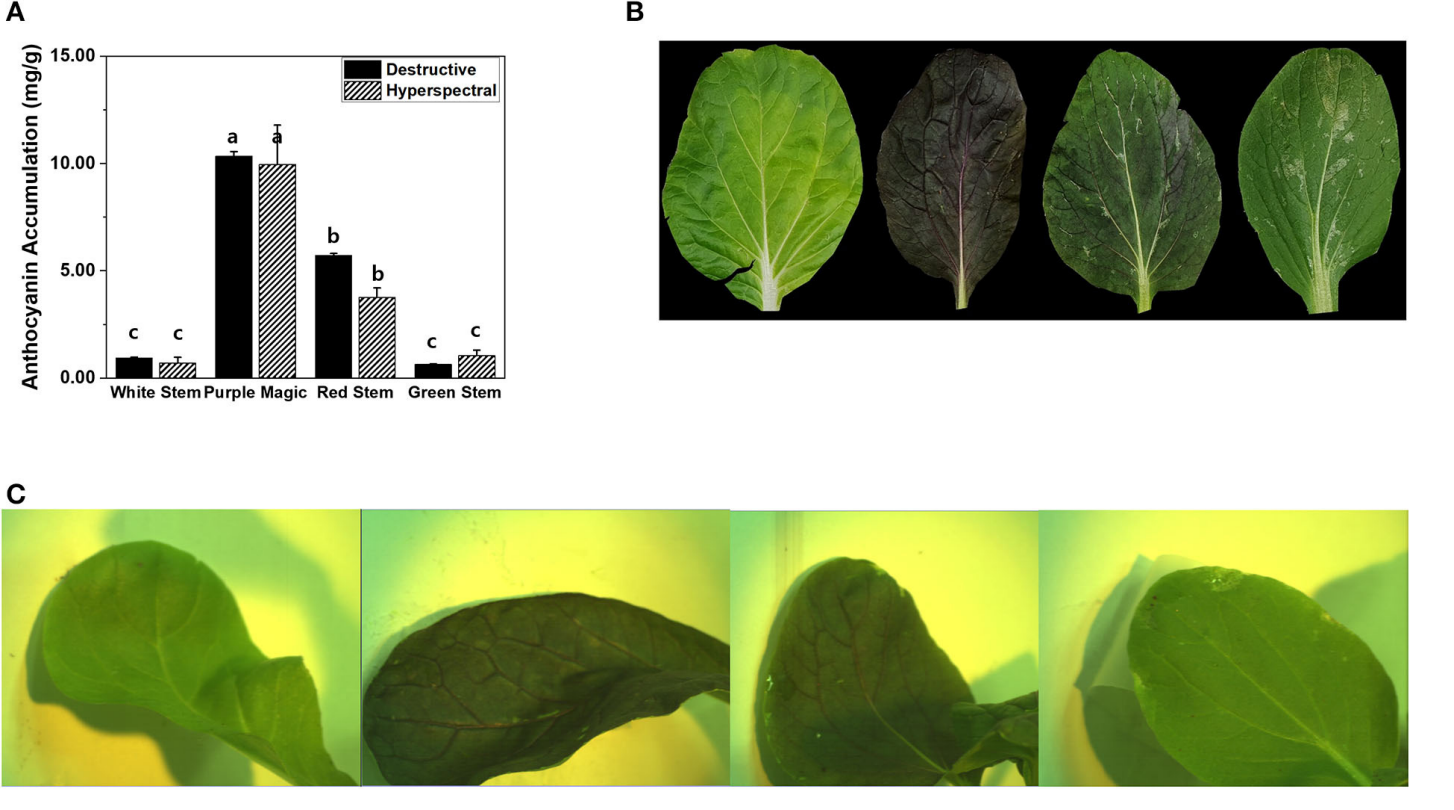
**(A)** Comparison of anthocyanin accumulations (mg/g DW) obtained by destructive analysis and hyperspectral analysis among targeted leaf of four bok choy cultivars grown under the SC at GS2. These data were obtained from the second experiment described in the Materials and methods section. Means with a different letter are significantly different at *P* ≦ 0.05, using Fisher's LSD analysis. **(B)** The representative image of four different bok choy cultivars (from the left white stem, purple magic, red stem, and green stem). **(C)** The representative hyperspectral image was taken in leaf-level in four different bok choy cultivars (from the left white stem, purple magic, red stem, and green stem).

Between the modified hyperspectral value and destructive analysis (UV) results, no significant differences in anthocyanin accumulation were observed across the four bok choy cultivars grown under the SC at GS2. Furthermore, the anthocyanin accumulations estimated by hyperspectral imaging and destructive analysis equally displayed cultivar-dependent significant differences across cultivars (Figure 2A). This result indicates that hyperspectral imaging can be employed to measure differences in variations of anthocyanin accumulations that occurred in the leaf tissue of different bok choy cultivars. Variations in anthocyanin accumulation in different cultivars seem to be related to the expression of color pigments in leaf tissues (Figure 2B). The “purple magic” showed the highest level of anthocyanin accumulation in both hyperspectral value and destructive analysis with the purple color expression in almost the whole area of the leaf, whereas “white stem” and “green stem” displayed the lowest level of anthocyanin accumulation with the no expression of purple color in a leaf. “red stem” showed a partial expression of purple color in the leaf tissue with the middle level of anthocyanin accumulation (Figure 2).

### Variations of Anthocyanin Accumulations Caused by Different Indoor Farm Cultivation Conditions

**Figure 3:**
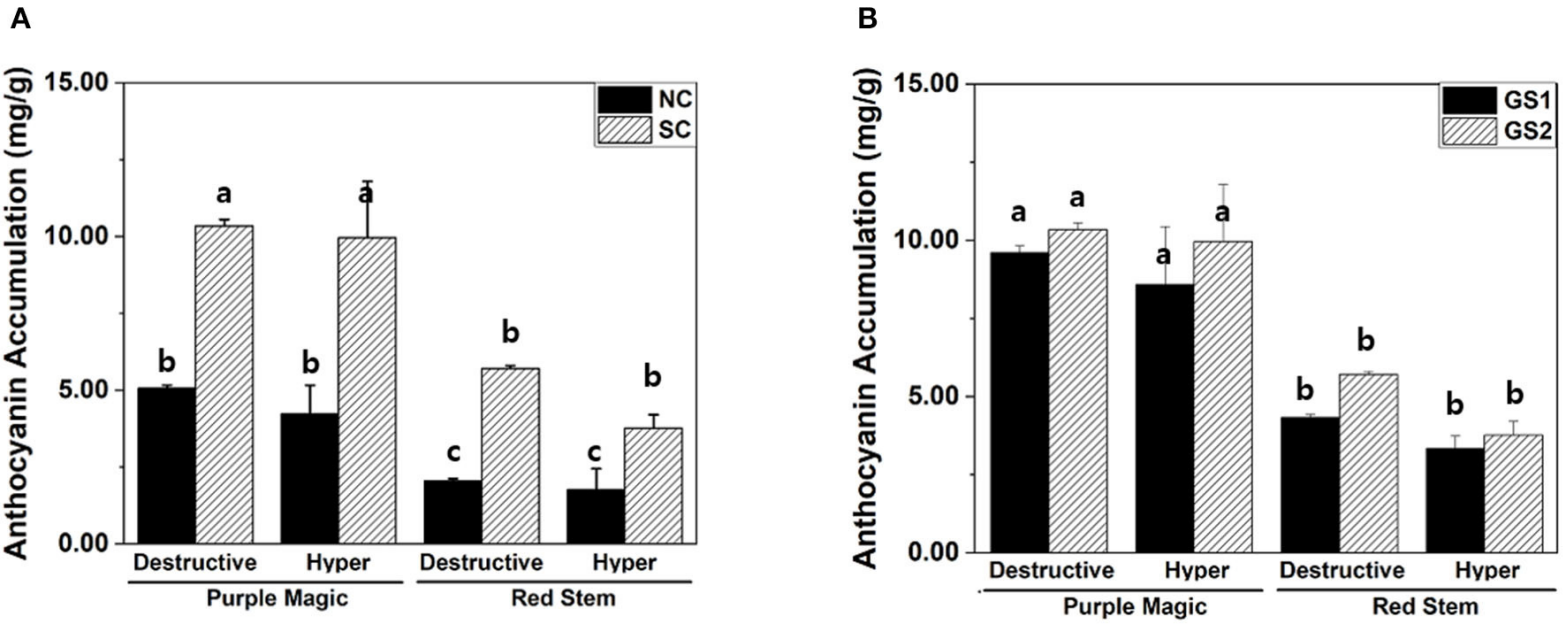
Variation in anthocyanin accumulations (mg/g DW) of purple magic and red stem estimated by different cultivation conditions. **(A)** Destructive and hyperspectral analysis with non-stimulation and stimulation conditions. **(B)** Destructive analysis and hyperspectral analysis by cultivation period for 2 weeks (GS1) and 4 weeks (GS2). These data were obtained from the second experiment described in the Materials and methods section. Means with a different letter are significantly different at *P* ≦ 0.05, using Fisher's LSD analysis.

Increased accumulation of anthocyanin under the SC was observed in “purple magic” and “red stem” by both hyperspectral imaging and destructive analysis (Figure 3A). In different growth stages (GS1 and GS2), no significant differences in anthocyanin accumulation between hyperspectral imaging and destructive analysis were found in each cultivar (Figure 3B). These results suggest that hyperspectral imaging can provide comparable analytic capability with destructive analysis to measure variations in anthocyanin accumulation under the different environmental conditions of the indoor farm. Although significant increases of anthocyanin accumulations were also found in “white stem” and “green stem” under the SC, these increases would be caused by the limit of analytic capability of both hyperspectral imaging and destructive analysis under the relatively lower level of anthocyanin compared with its level of “purple magic” and “red stem” (Table 1).

**Table 1:** Variation of anthocyanin accumulation (mg/g) in leaf tissue of four bok choy cultivars grown under the different environmental conditions (NC and SC) with different growth stages (GS1 and GS2).

**Genotype**	**Treatment^y^**	**TA (mg/g)^z^**		**TA (%)^z^**
		**Destructive (UV)^x^**	**Hyperspectral^x^**	**based on RGB color**
“White Stem”	NC-GS1	0.880 ± 0.023^g^	0.423 ± 0.027^f^	0.30 ± 0.20^f^
	SC-GS1	0.953 ± 0.043^g^	0.529 ± 0.080^e^	0.66 ± 0.36^f^
	NC-GS2	0.378 ± 0.030^h^	0.603 ± 0.051^e^	3.01 ± 1.11^e^
	SC-GS2	0.938 ± 0.048^g^	0.698 ± 0.263^e^	1.61 ± 0.79^ef^
“Purple Stem”	NC-GS1	6.848 ± 0.158^b^	1.982 ± 0.498^d^	17.08 ± 10.31^cde^
	SC-GS1	9.608 ± 0.218^a^	8.585 ± 1.860^b^	53.56 ± 11.62^b^
	NC-GS2	5.060 ± 0.103^cd^	4.232 ± 0.930^c^	54.21 ± 11.69^b^
	SC-GS2	10.3425 ± 0.215^a^	9.959 ± 1.836^a^	83.53 ± 4.20^a^
“Red Stem”	NC-GS1	1.160 ± 0.030^f^	0.550 ± 0.096^e^	0.82 ± 0.31^f^
	SC-GS1	4.325 ± 0.103^d^	3.344 ± 0.395^cd^	11.32 ± 4.38^de^
	NC-GS2	2.045 ± 0.070^e^	1.766 ± 0.678^d^	27.53 ± 9.44^c^
	SC-GS2	5.708 ± 0.098^c^	3.768 ± 0.436^c^	26.38 ± 8.53^cd^
“Green Stem”	NC-GS1	0.863 ± 0.030^g^	0.442 ± 0.009^f^	0.34 ± 0.09^f^
	SC-GS1	1.293 ± 0.093^f^	0.573 ± 0.128^e^	0.22 ± 0.06^f^
	NC-GS2	0.663 ± 0.030^gh^	0.620 ± 0.060^e^	5.64 ± 1.88^def^
	SC-GS2	0.633 ± 0.030^gh^	1.046 ± 0.255^d^	10.56 ± 2.60^de^

*Anthocyanin accumulation (mg/g) was analyzed by destructive analysis and hyperspectral imaging*.

z *TA: total anthocyanin accumulation*.

y *NC-GS1: treatment with low lights and slight temperature difference for 2 weeks, SC-GS1: treatment with high lights and extreme temperature difference for 2 weeks, NC-GS2: treatment with low lights and slight temperature difference for 4 weeks, SC-GS2: treatment with high light and extreme temperature difference for 4 weeks*.

x *Means within each column and cultivar with different letters are significantly different at P ≦ 0.05, using Fisher's LSD analysis*.

### Leaf Image Analysis-Based Estimation of Anthocyanin Accumulations

**Figure 4:**
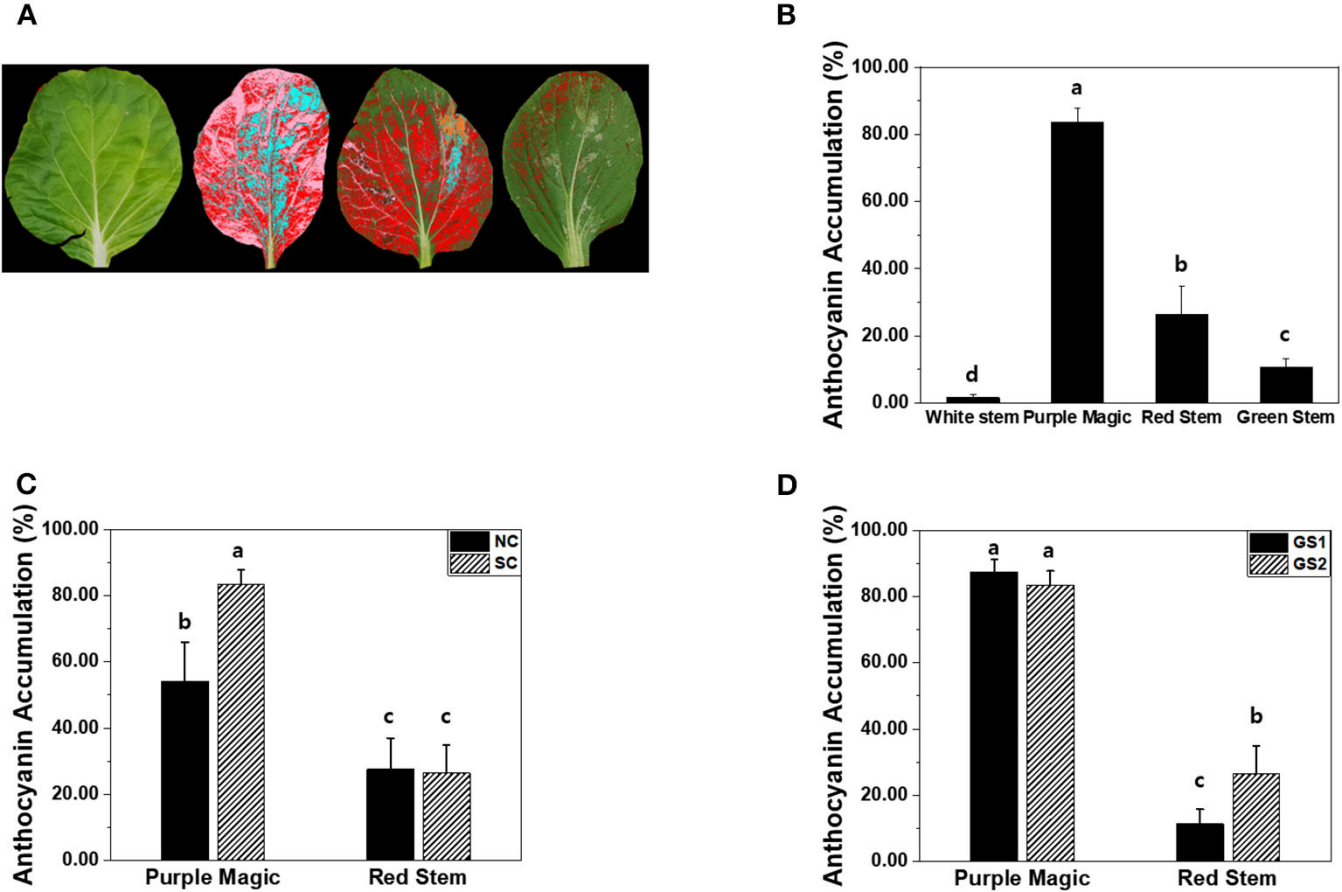
**(A)** The representative image of four different bok choy cultivars (from the left white stem, purple magic, red stem, and green stem) for leaf color analysis. **(B)** Comparison of anthocyanin accumulation (%) obtained by leaf color analysis among four cultivars, bok choy. **(C)** AT accumulation (%) of purple magic and red stem by non-stimulation (NS) and stimulation (CS) conditions. **(D)** AT accumulation (%) of purple magic and red stem by cultivation period for 2 weeks (GS1) and 4 weeks (GS2). These data were obtained from the second experiment described in the Materials and methods section. Means with a different letter are significantly different at *P* ≦ 0.05, using Fisher's LSD analysis.

Leaf image analysis is one of the potential approaches to estimate anthocyanin accumulations in a non-destructive manner. Unlike hyperspectral imaging and destructive analysis, leaf image analysis significantly increased anthocyanin accumulation in GS2 of “red stem” (Figures 3B, 4D). The percentages of the purple color area in the total leaf area among the four cultivars varied similar to the anthocyanin accumulation values measured by hyperspectral imaging and destructive analysis in the four cultivars (Figure 4B). However, leaf image analysis did not show a significant increase in anthocyanin accumulation in “red stem” under the SC compared with the results obtained by hyperspectral imaging and destructive analysis (Figures 3A, 4C). The different consequence was also found in anthocyanin accumulation of “red stem” leaves in different growth stages. These results indicated that the proportion of colored leaf area may be employed to estimate the anthocyanin accumulations of different cultivars showing clear differences in anthocyanin levels with quantitative values. However, it has limitations to reflect the level of color saturation caused by pigment accumulation, such as anthocyanin accumulation.

### Genotype and Environment Interaction

The ANOVA was applied to partition the variations in anthocyanin accumulation into components associated with the genotype, environment, and genotype × environment interaction (G × E) using the data obtained from hyperspectral imaging and destructive analysis, respectively (Table 2). Comparison of the results obtained from hyperspectral imaging and destructive analysis data demonstrated a similar proportion of genotypic and G × E effects with the same significance, indicating comparability of hyperspectral imaging with destructive analysis for the G × E study. The high proportion of anthocyanin variation was primarily contributed by the genotypic effect, indicating that selection and breeding of cultivars can be performed to optimize anthocyanin accumulation in bok choy for indoor farming. Since our data also demonstrated the significance of G × E interaction (Table 2), attention should be given to identifying germplasm in which anthocyanin biosynthesis is maximized in the specific environmental condition of indoor farms.

**Table 2:** Percentages of variations in anthocyanin accumulations (mg/g) associated with genotype, environment, and genotype by environment interaction for the leaves of four bok choy genotypes grown in different environmental conditions (NC and SC) with different growth stages (GS1 and GS2).

**Source of variation**	**Percentage of variations in TA^z^**	
	**Destructive (UV)**	**Hyperspectral**
Genotype	77.7*	59.2*
Environment	6.7	11.1
G × E ^y^	10.8**	26.6***
Residual	4.8***	2.9***

*Anthocyanin accumulation (mg/g) was analyzed by destructive analysis and hyperspectral imaging*.

z *TA: total anthocyanin accumulation*.

y *G × E = genotype × environment interaction*.

*, **, ***Significant at P ≦ 0.2, 0.05, or 0.025, respectively.

### Establishment of the Hyperspectral Imaging System to Be Applied to High-Throughput Plant Phenotyping System

**Figure 5:**
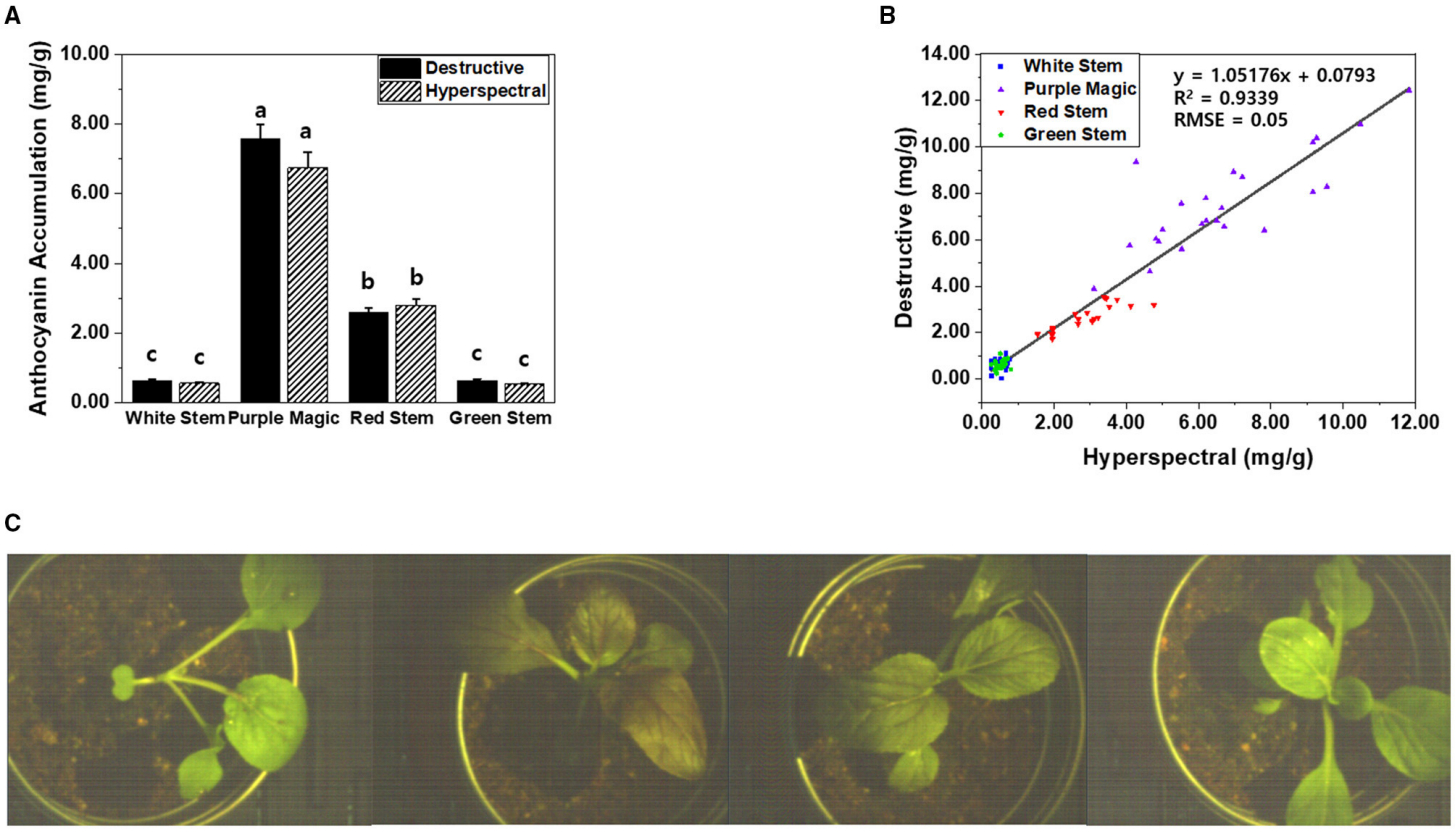
**(A)** Comparison of anthocyanin accumulations (mg/g DW) obtained by destructive analysis and hyperspectral analysis from the whole plant of four bok choy cultivars grown under the SC at GS1. These data were obtained from the third experiment described in the Materials and methods section. Means with a different letter are significantly different at *P* ≦ 0.05, using Fisher's LSD analysis. **(B)** The linear relationship between hyperspectral imaging and destructive analysis data was obtained from the whole plant of four bok choy cultivars grown under the SC at GS1. **(C)** The representative hyperspectral image was taken at the whole plant level in four different bok choy cultivars (from the left white stem, purple magic, red stem, and green stem).

In our previous study, a “sensor-to-plant” type plant phenotyping system was developed to accelerate the large-scale acquisition of plant images through the moving actuators with vision sensors in real-time (Lee et al., 2018). To apply our hyperspectral imaging onto the high-throughput phenotyping system, the hyperspectral imaging system was modified to acquire a top view image of the whole plant (Figures 1B, 5C). Two-week-old whole plants of four bok choy cultivars were subjected to hyperspectral imaging in the third experiment. Similarly, classified anthocyanin accumulations were observed across four cultivars in the results obtained from both hyperspectral imaging and destructive analysis (Figure 5A). In addition, the linear relationship between hyperspectral imaging and destructive analysis data (*R*^2^ = 0.9339, RMSE = 0.05) suggests that the hyperspectral imaging of the whole plant is comparable with the destructive analysis and feasible to be applied onto the high-throughput plant phenotyping system (Figure 5B).

## Discussion

There were various attempts to analyze anthocyanin accumulation non-destructively, such as the hyperspectral model (Gu et al., 2018), UV-Spec. (Gitelson et al., 2009), and the fluorimetric sensor (Tuccio et al., 2011). We tested previously established hyperspectral models, such as the ACI (Gitelson et al., 2006), ARI (Gitelson et al., 2001, 2006), and mARI (Gitelson et al., 2006) to select the optimum model to analyze anthocyanin accumulations using hyperspectral imaging in bok choy. We also attempted to optimize mARI to improve the measurement accuracy through the adjustment of wavelength in the first experiment, and we confirmed that our wavelength-adjusted mARI approach could improve the linear relationship (*R*^2^ = 0.9958) between destructive and non-destructive analyses for anthocyanin estimation. Further studies conducted with our optimized hyperspectral imaging approach in the second and third experiments demonstrated comparable trends in changes of anthocyanin accumulations across different cultivars and different environmental conditions between hyperspectral imaging and destructive analysis, although statistical significance was limited in the cultivars and environmental conditions inducing a relatively lower level of anthocyanin accumulation.

High-throughput phenotyping has received considerable attention due to its potential for rapid identification of valuable germplasm (Matsuda et al., 2012; Banerjee et al., 2020). In our previous study, we established a high-throughput phenotyping system consisting of an image-capturing hardware module, environmental data sensors, and automated irrigation and artificial light controllers (Lee et al., 2018). In this system, a total of 28 plant trays (52.5 cm × 26.5 cm in size) are placed in a 4 × 7 matrix, and the image acquisition module moves over 28 plant trays following the pre-defined X, Y, and Z coordinates of the plant trays. In each plant tray, 8 of 10 cm pots are placed. Therefore, a total of 280 pots can be placed in our phenotyping system. Our hyperspectral imaging system was modified to be applied to our high-throughput phenotyping system (Figure 1B). Using our hyperspectral imaging system, it took 2 min to acquire a top view image of the whole plant in 10 cm pots, and an additional 1 min to move pots and process data. It indicated that a total of 280 plants in 10 cm pots could be scanned by hyperspectral imaging under our phenotyping system for 14 h for rapid identification of bok choy germplasms.

In the conventional indoor farm, increasing artificial light intensity and day/night temperature differences to improve anthocyanin accumulation in vegetables also may cause an increase in the energy costs. To solve this conflicting problem, not only selection of proper germplasm that can show a better performance of anthocyanin accumulation under the acceptable indoor farming environments but also identification of optimum environmental conditions through the monitoring of changes in anthocyanin accumulations under the diverse indoor farm environmental conditions need to be accomplished as a further study. According to the previous studies, lettuce grown under 5°C of night-time temperatures showed a relatively higher polyphenolic content than lettuce cultivated under 20°C for 5 days. However, phenol content showed the highest levels at 20°C in 20 days of cultivation duration (Jeong et al., 2015). In another study, anthocyanin, carotenoid, and relative chlorophyll contents were decreased when purple bok choy was exposed to low light (250 μmol/m^2^/s) compared with high light (1,000 μmol/m^2^/s) (Zhu et al., 2017). These reports suggest that additional studies on the change of anthocyanin accumulations in bok choy with more diverse environmental conditions for more than 4 weeks of cultivation period using hyperspectral analysis are considered to be necessary. We believe that hyperspectral imaging can be an affordable approach to perform it in a non-destructive manner.

## Data Availability Statement

The original contributions presented in the study are included in the article/Supplementary Material, further inquiries can be directed to the corresponding author/s.

## Author Contributions

H-sK and JY performed the experiment, data collection, statistical data analysis, presentation, and drafted the manuscript. SP, J-SK, and YC experimented and analyzed the data. JK and HK conceived the project and experimental design and collaborated on the data interpretation and manuscript revision. All authors contributed to the article and approved the submitted version.

## Conflict of Interest

The authors declare that the research was conducted in the absence of any commercial or financial relationships that could be construed as a potential conflict of interest.

## Publisher's Note

All claims expressed in this article are solely those of the authors and do not necessarily represent those of their affiliated organizations, or those of the publisher, the editors and the reviewers. Any product that may be evaluated in this article, or claim that may be made by its manufacturer, is not guaranteed or endorsed by the publisher.

## Supplementary Material

Supplementary Figure 1 Reflectance index model for estimation of anthocyanin accumulation between the destructive (UV) and non-destructive (Hyperspectral), mARI of R706.35 **(A)**, mARI of R701.06 **(B)**, abs **(C)**, ARI **(D)**, and CI **(E)**.
