# Supplementary figures and images for: Measurement of Environmentally Influenced Variations in Anthocyanin Accumulations in Brassica rapa subsp. Chinensis (Bok Choy) Using Hyperspectral Imaging
Source: Front Plant Sci. 2021 Aug 19;12:693854. doi: 10.3389/fpls.2021.693854 (PMC8416915; doi:10.3389/fpls.2021.693854)

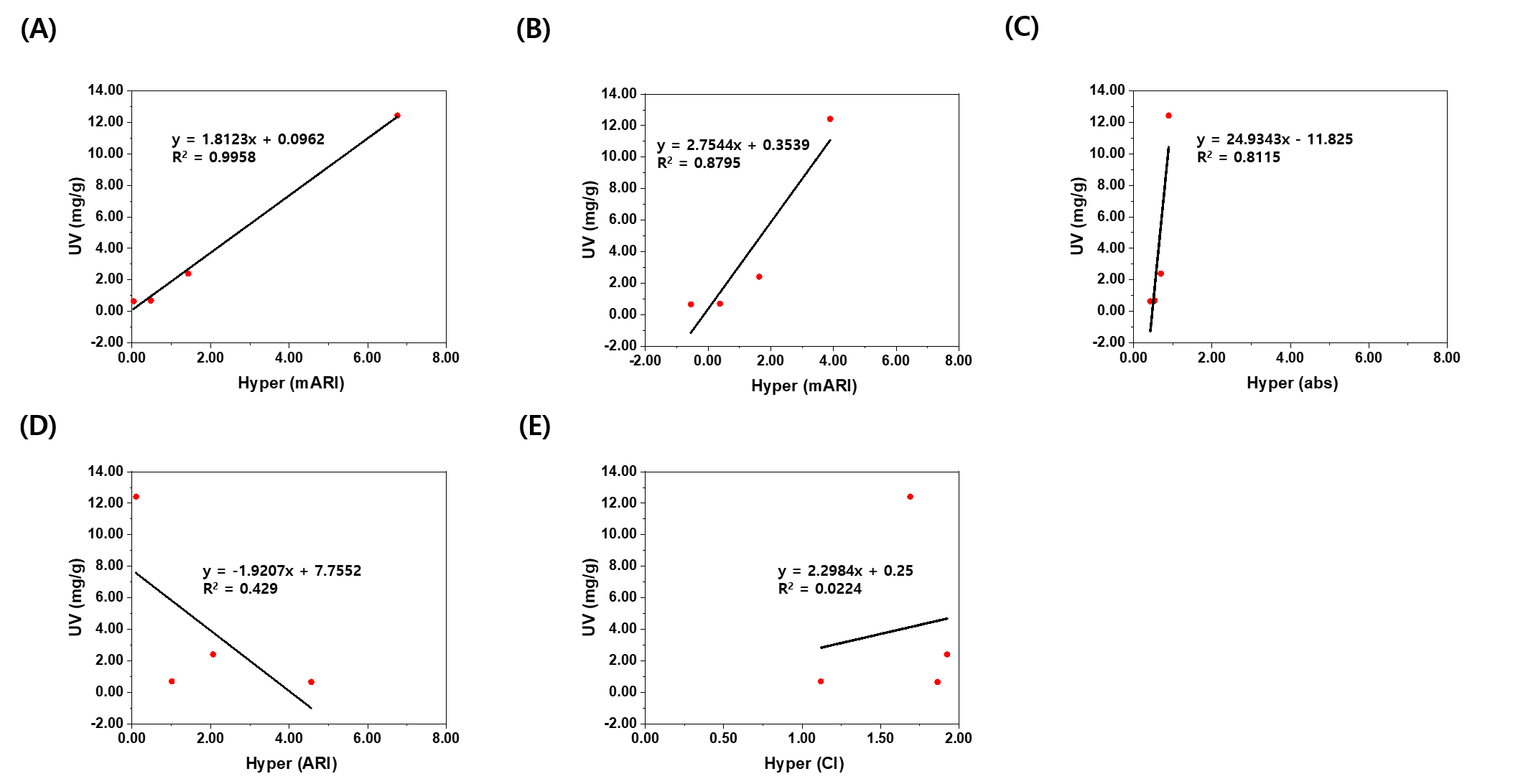

Supplement: Supplementary Figure 1 — Reflectance index model for estimation of anthocyanin accumulation between the destructive (UV) and non-destructive (Hyperspectral), mARI of R706.35 (A), mARI of R701.06 (B), abs (C), ARI (D), and CI (E). [file Image_1.TIF]
